# First record of *Trypanosoma evansi* DNA in *Dichelacera alcicornis* and *Dichelacera januarii* (Diptera: Tabanidae) flies in South America

**DOI:** 10.1186/s13071-022-05562-7

**Published:** 2023-01-05

**Authors:** Carlos José Raupp Ramos, Cintia de Souza Franco, Sabrina Pogere da Luz, Júlia Marques, Ketriane Mota de Souza, Luiz Flávio Nepomuceno do Nascimento, Gabriella Bassi das Neves, Renato Simões Moreira, Luiz Claudio Miletti

**Affiliations:** 1 Laboratório de Bioquímica de Hemoparasitas e Vetores, Departamento de Produção Animal e Alimentos, Centro de Ciências Agroveterinárias, Universidade do Estado de Santa Catarina, Avenida Luiz de Camões, Lages, SC 2090 Brazil; 2 Universidade Federal da Fronteira Sul, Campus Laranjeiras do Sul, Rodovia BR 158—Km 405, Laranjeiras do Sul, PR 85301-970 Brazil; 3 Instituto Federal de Santa Catarina (IFSC), Campus Lages, Rua Heitor Villa Lobos 222, São Francisco, Lages, SC 88506-400 Brazil

**Keywords:** Tabanidae, Vectors, Transmission, Polymerase chain reaction, *Trypanosoma*

## Abstract

**Background:**

*Trypanosoma evansi* infects a large number of wild and domestic animals and causes a spoliative disease known as surra. It is mechanically transmitted, mainly by biting flies of the genera *Tabanus* and *Stomoxys*. The detection of *T. evansi* DNA in the feeding apparatus of *Dichelacera alcicornis* and *Dichelacera januarii* from South America is reported, to the best of our knowledge, for the first time.

**Methods:**

Tabanids were collected weekly from February 2018 to February 2019 from two sites. The feeding apparatus was removed and DNA extraction, polymerase chain reaction and sequencing were performed.

**Results:**

A 205-base pair fragment of the variant surface protein RoTat 1.2 gene, confirmed by DNA sequencing, was amplified from the feeding apparatus of *D. alcicornis* and *D. januarii.*

**Conclusions:**

This is, to the best of our knowledge, the first record of *T. evansi* DNA in South American tabanids.

**Graphical abstract:**

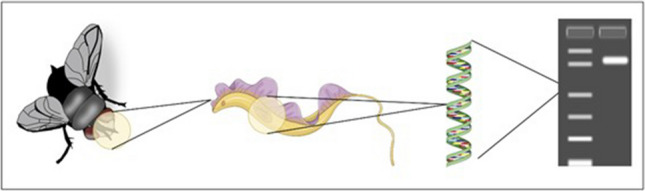

**Supplementary Information:**

The online version contains supplementary material available at 10.1186/s13071-022-05562-7.

## Background

The family Tabanidae, which belongs to the order Diptera, comprises approximately 4400 species [1]. These insects are of great medical and veterinary interest due to their persistent and irritating bites and their ability to transmit 32 pathogens, including viruses, bacteria, helminths, and protozoa, such as *Trypanosoma* spp. [1].

*Trypanosoma evansi* (Steel, 1885) is the etiological agent of surra, a disease that affects a variety of domestic and wild mammals. *Trypanosoma evansi* is distributed in Africa, Asia, and South America and causes important economic losses to livestock farming. Surra is of great concern because of the absence of pathognomonic signs, including fever, anemia, loss of body weight, low production of animal protein, nervous signs, abortion, cachexia, and death, with or without more particular signs related to the host species [2]. *Trypanosoma evansi*, which is mechanically transmitted by tabanids and is widespread in regions of Brazil and the rest of South America, causes outbreaks of surra in horses [3].

The focus of most of the published literature on tabanid species from Brazil and the rest of South America is their taxonomy [4–7], ecology [8, 9] and behavior [10, 11] in different biomes, and molecular tools were not used for identification or bioprospecting in these studies. More specifically, few studies have addressed tabanids in southern Brazil [12–17], and only one reported the molecular detection of a strain of a trypanosomatid, *Trypanosoma kaiowa*, which is associated with the crocodilian clade of Trypanosoma [18]. Cases of trypanosomosis caused by *T. evansi* [19] in that region have been described in which the involvement of tabanids was assumed. There are no data on the detection of *T. evansi* in tabanid flies in South America. This is, to the best of our knowledge, the first study to describe the molecular detection of *T. evansi* DNA in *Dichelacera alcicornis* (Wiedemann, 1828) and *Dichelacera januarii* (Wiedemann, 1819), both of which are widely distributed in various biomes of South America [20] and may be related to outbreaks of surra.

## Methods

### Study site

This study was carried out in the municipality of Lages (27°48′57″S, 50°19′33″ W), Santa Catarina State, Brazil, which has a mixed rainforest terrain and altitude of 930 m above mean sea level.

The Köppen climate classification of the municipality is Cfb, i.e. it has a temperate oceanic climate. The average temperature in the coldest month is above 0 °C, the average annual temperature is below 22 °C, and at least 4 months have an average temperature above 10 °C, with no significant difference in precipitation between the seasons [21].

### Sample collection

Tabanids were collected weekly from February 2018 to February 2019 from two rural properties located in different parts of the region in which especially cattle and horses are reared.

The collections were made once a week at the same location on each property, close to lakes and rivers, for 3 h, from 3 p.m. until 6 p.m., using a contained horse from each property as bait throughout the collection period. Tabanids that landed on the horses were carefully captured with a glass tube (4.5 cm diameter, 9 cm length) [14]. The flies were placed in individual plastic bottles using a SECTAB device [22]. The collected insects were transported to the Laboratory of Hemoparasites and Vectors (Lages, Santa Catarina, Brazil) and killed with chloroform in flasks.

### Taxonomic identification of tabanids

Taxonomic identification was performed according to the taxonomic keys described by Fairchild and Philip [23], and Dr. Inocêncio de Sousa Gorayeb (Museu Paraense Emílio Goeldi, Belém, Brazil) verified the identification of the species. The collected specimens were deposited at the Entomology Museum of the Federal University of Fronteira Sul, Laranjeiras do Sul campus, Paraná, Brazil.

### DNA extraction

The extraction of DNA from the mouthparts was initially carried out for the three most abundant species of tabanid. After these analyses, DNA was also extracted from the other species of *Dichelacera* captured, *D. januarii*.

The flies were washed twice with 70% ethanol solution and twice with sterile distilled water. The mouthparts were removed with the aid of sterile fine scissors under an entomological magnifying glass. The mouthparts were stored in 1.5-mL microtubes in Tris–NaCl–ethylenediaminetetraacetic acid (ETDA) buffer (10 mM Tris base, 200 mM NaCl and 50 mM EDTA), and stored frozen at -80 °C until DNA extraction.

For DNA extraction, the mouthparts were placed in a 1.5-mL microtube and macerated with an appropriate sized pistil. The DNA sample was extracted once with phenol (pH 7.8), once with phenol:chloroform (1:1), and once with chloroform:isoamyl alcohol (24:1). The DNA of the sample was then precipitated using sodium acetate (pH 6.0) and absolute ethanol and resuspended in 50 µL Tris–EDTA buffer (10 mM Tris–HCl, 1 mM EDTA) [24].

### Polymerase chain reaction amplification

Flies were individually screened for the presence of *Trypanosoma evansi* and *Trypanosoma vivax* using specific oligonucleotide primers, RoTat 1.2 forward 5′GCGGGGTGTTTAAAGCAATA3′ and RoTat 1.2 reverse 5′ATTAGTGCTGCGTGTGTTCG3′ for *T. evansi* [24], and TviSL1 5′GCTCTCCAATCTTAACCCTA3′ and TviSL2 5′GTTCCAGGCGTGCAAACGTC3′ for *T. vivax* [25]. The first two primers amplified a 205-base pair (bp) fragment and the second two a 210-bp fragment. Polymerase chain reaction (PCR) was conducted in a 400-µL reaction mixture comprising 323.5 µL deionized water, 10.5 µL MgCl_2_, 1.75 µL Taq DNA polymerase, 7 µL deoxyribonucleotide triphosphate mix (10 mM), 35 µL of 10× Taq DNA polymerase buffer, 10.5 µL of each forward primer, 7 µL of each reverse primer, and 5 µL of the template. *Trypanosoma evansi* DNA was obtained from parasites purified from the blood of experimentally infected albino rats. *Trypanosoma vivax* DNA was obtained from purified parasites of experimentally infected sheep (approved by the Animal Experimentation Ethics Committee of Universidade do Estado de Santa Catarina) and was used as a positive control. Nuclease-free water was added to the PCR mix instead of a DNA sample as a negative control.

PCR was performed using an automated DNA thermal cycler (Biocycler). The amplification conditions were: initial denaturation at 94 °C for 3 min followed by 35 denaturation cycles at 94 °C for 30 s, annealing at 62 °C for 30 s, primer extension at 72 °C for 1 min, and a final extension at 72 °C for 4 min. The final phase of the PCR included cooling the samples to 10 °C. The PCR products were visualized on a 1% agarose gel stained with ethidium bromide.

### Sequencing and Basic Local Alignment Search Tool

The PCR amplicons were purified using the QIAGEN Gel Purification Kit (QIAGEN, Hilden, Germany) according to the manufacturer’s protocol. Sequencing was conducted using the BigDye Terminator Cycle Sequencing Kit according to the manufacturer’s protocol (Applied Biosystems, Carlsbad, CA). The eluent was loaded into a 96-well plate which was placed into an ABI Prism 3500 Genetic Analyzer (Applied Biosystems).

Each DNA sample was purified according to the following protocol: 50 µL sample DNA was added to a mixture containing 5 µL of 3 M sodium acetate, 125 µL of 100% ethanol, and 2 µL glycogen (20 mg/ml) and placed in a freezer at − 80 °C for 1 h. Following centrifugation at 12,000 *g* for 45 min at 4 °C, the pellet formed was washed once with 75% ethanol and centrifuged for another 15 min at 75,000 *g* at 4 °C. The mixture was then dried in a SpeedVac at 20–25 °C for 30 min and resuspended in 20 µL ultrapure water (Milli-Q).

The retrieved gene sequences were edited using BioEdit software [26]. The nucleotide Basic Local Alignment Search Tool (BLASTn) was used (www.ncbi.nlm.nih.gov/blast/) to confirm the sequences obtained from the PCR analysis. Gene sequences with match scores of 80–100% similarity were considered significant.

## Results

A total of 523 female tabanids were collected from February 2018 to February 2019, specifically in February, March, April, November and December 2018, and January and February 2019. There was no evidence of tabanids in the other months of the collection period.

Individuals of 14 species and seven genera were collected (Table 1). *Dichelacera alcicornis* was the most abundant species, representing 52.77% (276) of the total, followed by *Chrysops fusciapex* (Lutz, 1909a) (17.97%, 94) and *Chrysops patricia* (Pechuman, 1953) (10.70%, 56), similar to the results of previous studies [11, 14]. The least abundant species were *Tabanus fuscus* (Wiedemann, 1819), *Tabanus nebulosus* (De Geer, 1776) and *Acanthocera kroeberi* (Fairchild, 1939), and represented 0.19% of the total.

**Table 1:** Tabanid species and numbers of individuals caught from February 2018 to February 2019 in Lages, Santa Catarina, Brazil

Species	No. of individuals	Percentage (%)
*Dichelacera alcicornis*	276	52.77
*Chrysops fusciapex*	94	17.97
*Chrysops patricia*	56	10.70
*Poeciloderas quadripunctatus*	23	4.39
*Fidena nigripes*	17	3.25
*Fidena longipalpis*	15	2.86
*Tabanus colombensis*	15	2.86
*Dichelacera januarii*	12	2.29
*Acanthocera aureoscutellata*	5	0.95
*Catachlorops* sp*.*	5	0.95
*Tabanus* sp*.*	2	0.38
*Acanthocera kroeberi*	1	0.19
*Tabanus fuscus*	1	0.19
*Tabanus nebulosus*	1	0.19
Total	523	100

**Fig. 1:**
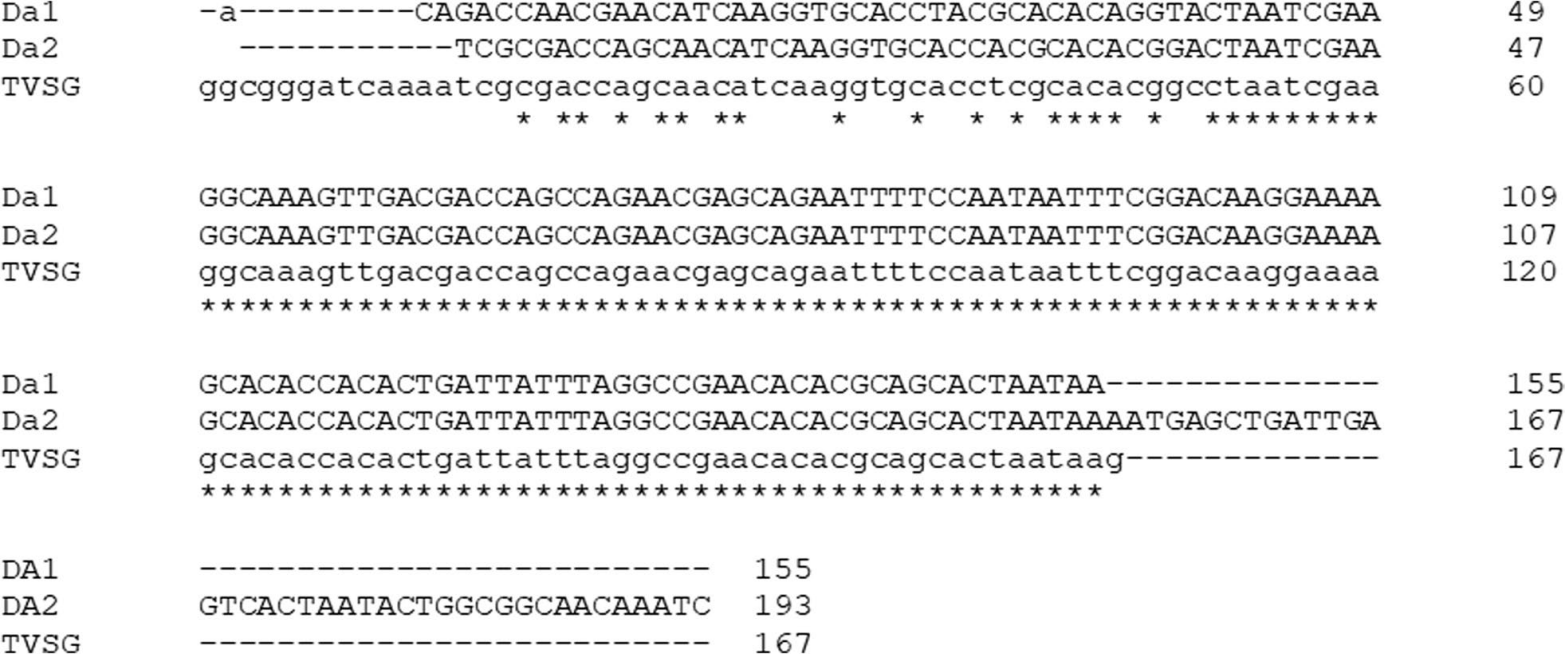
Multiple sequence alignment between the polymerase chain reaction amplified sequences of DNA from the feeding apparatus of *Dichelacera alcicornis* (Da1) and *Dichelacera januarii* (Da2) and the variant surface glycoprotein sequence of *T. evansi* deposited in GenBank

Samples of the three most abundant species, *D. alcicornis* (Additional file 1: Fig. 1), *C. patricia* and *C*. *fusciapex*, were analyzed. Samples of *D*. *januarii* (Additional file 2: Fig. 2) were also examined so that both species of *Dichelacera* that had been captured were included in the analyses. The PCR amplicons were positive for *T. evansi* using the 205-bp RoTat 1.2 gene. Sequencing and analysis of the amplicons demonstrated that the sequence obtained corresponded to the *T. evansi* variable surface glycoprotein (accession no. MZ209177.1). It was present in the feeding parts of *D. alcicornis* (Da1) with 96% identity at site 1 and in *D. alcicornis* and *D. januarii* (Da 2) with 99% identity at site 2 (Fig. 1). There was no amplification of the *T. vivax* splice leader gene for any of the samples. The sequences were deposited in GenBank (respective accession numbers OM971942 and OM971943).

For *C. patricia* and *C*. *fusciapex* there was no amplification of the *T. evansi* RoTat 1.2 gene or *T. vivax* TviSL gene. The presence of DNA of other trypanosomatids was not evaluated due to the focal objective of the work.

## Discussion

The few studies that exist on the seasonality of the Tabanidae in southern Brazil show *D. alcicornis* to be one of the most abundant species of this family in the region [6, 9, 10], as also found in the present study. The circulation of *T. evansi* in southern Brazil has already been demonstrated in several studies [14, 27–29], but this is the first one, to the best of our knowledge, in which the DNA of this parasite has been detected in the mouthparts of members of the Tabanidae. The sequenced PCR products showed high identity with the deposited sequences in GenBank, which is considered evidence of the presence of *T. evansi* DNA in the mouthparts of the tabanids examined here.

Two recent studies carried out in Brazil (in the Pantanal region and on the Coastal Plain of the state of Rio Grande do Sul) identified *Trypanosoma kaiowa* in tabanids using an insect dissection method and the observation of internal organs under an optical microscope [30] and through molecular detection [18]. Protozoan parasites have been detected in tabanids from other continents, such as Africa (particularly South Africa and Zambia), and included *Trypanosoma congolense*, *Trypanosoma theileri*, and *Trypanosoma evansi* [31]. In Europe (Poland), four species of tabanids (*Haematopota pluvialis*, *Tabanus bromius*, *Tabanus maculicornis*, and *Tabanus distinguendus*) were found to be infected by trypanosomatids of the subgenus *Megatrypanum*, of which *Trypanosoma theileri* is a member, and the occurrence of a trypanosomatid in *T. maculicornis* and *T. distinguendus* was described in that study for the first time [32]. *Chrysops laetus* and *Dichelacera tetradelta* infected with *T. theileri* were recently described for northern Brazil [33]. Our data, are, to the best of our knowledge, the first molecular confirmation of the presence of *T. evansi* DNA in the feeding apparatus of *D. alcicornis* and *D. januarii*, and support previous findings.

Although reservoirs play a central role in the maintenance and expansion of *T. evansi* infections, transmission depends on several ecological and epidemiological characteristics, such as the presence of competent vector species and susceptible mammalian hosts. To the best of our knowledge, there have been no studies on the vectorial capacity of the genera and species examined in the present study. However, a study has demonstrated the transmission of *T. evansi* by species of the genus *Stomoxys* under laboratory conditions [34]. A mathematical model has been developed that demonstrates the conditions necessary for the successful mechanical transmission of pathogens by tabanids [35]. Thus, the development and use of molecular detection approaches could help improve the identification of disease-causing agents and their tabanid vectors, in addition to facilitating the mapping of the circulation of these agents.

## Conclusion

This is, to the best of our knowledge, the first report of *T. evansi* DNA in South American tabanids.

## Supplementary Information

**Additional file 1: Figure S1.** General morphology (a) and detail of head (b), wings (b), and antenna (d) of *Dichelacera alcicornis.* The scales are not the same in this composite figure.

**Additional file 2: Figure S2.** General morphology (a) and detail of head (b), wings (c), and antenna (d) of *Dichelacera januarii*. The scales are not the same in this composite figure.

## Data Availability

The datasets used or analyzed during the current study are available from the corresponding author on reasonable request.

## Abbreviations

PCR: Polymerase chain reaction
EDTA: Ethylenediaminetetraacetic acid

## References

[1] Baldacchino F, Desquesnes M, Mihok S, Foil LD, Duvallet G, Jittapalapong G (2014). Tabanids: neglected subjects of research, but important vectors of disease agents. Infect Gen Evol.

[2] Desquesnes M, Holzmuller P, Lai DH, Dargantes A, Lun ZR, Jittaplapong S (2013). *Trypanosoma evansi* and surra: a review and perspectives on origin, history, distribution, taxonomy, morphology, hosts, and pathogenic effects. Biomed Res Int.

[3] Büscher P, Gonzatti MI, Hébert L, Inoue N, Pascucci I, Schnaufer A (2019). Equine trypanosomosis: enigmas and diagnostic challenges. Parasite Vectors.

[4] Henriques Al, Krolow TK, Zaarchi TBO, Camargo LMA (2022). Description of *Tabanus rondoniensis* (Diptera: Tabanidae), a new species of horsefly from the state of Rondônia, Brazil. Biodivers Data J.

[5] Lima HIL, Krolow TK, Henriques AL (2018). A new species of* Dichelacera* (*Dichelacera*) Macquart (Diptera, Tabanidae) from the Brazilian savannah. Neotrop Entomol.

[6] Guimarães RR, Gorayeb IS, Rodrigues-Guimarães R, Seppa GS, Carvalho RW (2015). Description of *Dichelacera* (*Dichelacera*) *walteri* n. sp. (Diptera: Tabanidae) with a key to related species in the subgenus* Dichelacera* Macquart. Neotrop Entomol.

[7] Krolow TK, Lucas M, Henriques AL (2022). Revisiting the tabanid fauna (Diptera: Tabanidae) of Uruguay: notes on the species of the genus* Tabanus* Linnaeus, with the description of a new species. Neotrop Entomol.

[8] Yamazaki A, Suganuma K, Kayano M, Acosta TJ, Saitoh T, Valinotti MFR (2022). Risk factors for equine trypanosomosis and hematological analysis of horses in Paraguay. Acta Trop.

[9] Ferreira RLM, Henriques AL, Rafael JA (2002). Activity of tabanids (Insecta: Diptera: Tabanidae) attacking the reptiles *Caiman crocodilus* (Linn.) (Alligatoridae) and *Eunectes murinus* (Linn.) (Boidae), in the central Amazon, Brazil. Mem Inst Oswaldo Cruz.

[10] Barros ATM (2001). Seasonality and relative abundance of Tabanidae (Diptera) captured on horses in the Pantanal, Brazil. Mem Inst Oswaldo Cruz.

[11] Bassi RMA, Cunha MCI, Coscaron S (2000). A study of behavior of tabanids (Diptera, Tabanidae) from Brazil. Acta Biol Par.

[12] Krolow TK, Krüger RF, Ribeiro PB (2007). Illustrated key for Tabanidae (Insecta: Diptera) genera of Campos Sulinos biome, Rio Grande do Sul. Brazil Bio Neotrop.

[13] Turcatel M, Carvalho CJB, Rafael JA (2007). Horseflies (Diptera: Tabanidae) of Paraná state, Brazil: pictorial identification key for subfamilies, tribes and genera. Bio Neotrop.

[14] Miletti LC, Colombo BB, Cardoso CP, Stalliviere FM, Tavares KCS, Komati LKO (2011). Prevalence, seasonality, and behavior of Tabanidae (Diptera) captured on a horse in the Planalto Serrano of Santa Catarina State, Brazil. Int J Trop Insect Sci.

[15] Dutra RRC, Marinoni RC (1994). Insetos capturados com armadilha malaise na Ilha do Mel, Baia de Paranágua, Paraná, Brasil. II. Tabanidae (Diptera). Rev Bras Zool.

[16] Bassi RMA (1997). Descrição de *Fidena campolarguense* sp. n. (Diptera, Tabanidae) do Brasil. Acta Biol Par.

[17] Kruger RF, Krolow TK (2015). Seasonal patterns of horse fly richness and abundance in the Pampa biome of southern Brazil. J Vet Ecol.

[18] Rodrigues GD, Blodorn E, Zafalon-Silva A, Domingues W, Marques R, Krolow TK (2022). Molecular detection of *Trypanosoma kaiowa* in *Tabanus triangulum* (Diptera: Tabanidae) from the coastal plain of Rio Grande do Sul, southern Brazil. Acta Parasitol.

[19] Reck C, Menin Á, Pisetta NL, Batista F, Miletti LC (2020). First outbreak of autochthonous “surra” in horses in Santa Catarina State, Brazil: parasitological, hematological and biochemical characteristics. Vet Parasitol Reg Stud Rep.

[20] Coscarón S, Papavero N (2009). Manual of Neotropical Diptera. Tabanidae Neotrop Diptera.

[21] Peel MC, Finlayson BL, McMahon TA (2007). Updated world map of the Köppen–Geiger climate classification. Hydrol Earth Syst Sci.

[22] Christen S, Tavares KCS, Komati LKO, Ramos CJR, Miletti LC (2009). SECTAB—a new device for tabanid storage in field collections. Neotrop Entomol.

[23] Fairchild GB, Philip CB (1960). A revision of the Neotropical genus *Dichelacera* subgenus *Dichelacera*, Macquart (Diptera, Tabanidae). Studia Entomol SP.

[24] Claes F, Radwanska M, Urakawa T, Majiwa PA, Goddeeris B, Büscher P (2004). Variable surface glycoprotein RoTat 1.2 PCR as a specific diagnostic tool for the detection of *Trypanosoma evansi* infections. Kinet Biol Dis.

[25] Ventura RM, Paiva F, Silva RA, Takeda GF, Buck GA, Teixeira MM (2001). *Trypanosoma vivax*: characterization of the spliced-leader gene of a Brazilian stock and species-specific detection by PCR amplification of an intergenic spacer sequence. Exp Parasitol.

[26] Hall TA (1999). BioEdit: a user-friendly biological sequence alignment editor and analysis program for Windows 95/98/NT. Nucleic Acids Symp Ser.

[27] Rodrigues A, Fighera RA, Souza TM, Schild AL, Soares MP, Milano J (2005). Outbreaks of trypanosomiasis in horses by *Trypanosoma evansi* in the state of Rio Grande do Sul, Brazil: epidemiological, clinical, hematological, and pathological aspects. Pesq Vet Bras.

[28] Silva SS, Oliveira CB, Zanette RA, Soares CDM, Coradini G, Polenz CH, et al. Ocorrência de *Trypanosoma evansi* em bovinos de uma propriedade leiteira no município de Videira—SC, Brasil. Acta Sci Veter. 2007;35:373–376. 10.22456/1679-9216.16133

[29] Zanette RA, Silva AS, Costa M, Monteiro SG, Santurio JM, Lopes STA (2008). Ocorrência de *Trypanosoma evansi* em eqüinos no município de Cruz Alta, RS, Brasil. Ciência Rural.

[30] Fermino BR, Paiva F, Viola LB, Rodrigues CMF, Garcia HA, Campaner M (2019). Shared species of crocodilian trypanosomes carried by tabanid flies in Africa and South America, including the description of a new species from caimans, *Trypanosoma kaiowan* sp. Parasit Vectors.

[31] Taioe MO, Motloang MY, Namangala B, Chota A, Molefe NI, Musinguzi SP (2017). Characterization of tabanid flies (Diptera: Tabanidae) in South Africa and Zambia and detection of protozoan parasites they are harboring. Parasitol.

[32] Werszko J, Szewczyk T, Steiner-Bogdaszewska Ż, Wróblewski P, Karbowiak G, Laskowski Z (2020). Molecular detection of *Megatrypanum* trypanosomes in tabanid flies. Med Vet Entomol.

[33] Bilheiro AB, Camargo JSAA, Zamaechi TBO, Tonholo C, Bassin HCM, Sussuarana ITA (2019). Survey of* Trypanosoma* (Kinetoplastida: Trypanosomatidae) infection in Monte Negro municipality, state of Rondônia, Western Amazon, with first record of *T. evansi* in the state. Rev Soc Bras Med Trop.

[34] Sumba AL, Mihok S, Oyieke FA (1998). Mechanical transmission of *Trypanosoma evansi* and *T. congolense* by *Stomoxys niger* and *S. taeniatus* in a laboratory mouse model. Med Vet Entomol.

[35] Desquesnes M, Biteau-Coroller F, Bouyer J, Dia ML, Foil L (2009). Development of a mathematical model for mechanical transmission of trypanosomes and other pathogens of cattle transmitted by tabanids. Int J Parasitol.

