# Supplementary figures and images for: First record of Trypanosoma evansi DNA in Dichelacera alcicornis and Dichelacera januarii (Diptera: Tabanidae) flies in South America
Source: Parasit Vectors. 2023 Jan 5;16:4. doi: 10.1186/s13071-022-05562-7 (PMC9817266; doi:10.1186/s13071-022-05562-7)

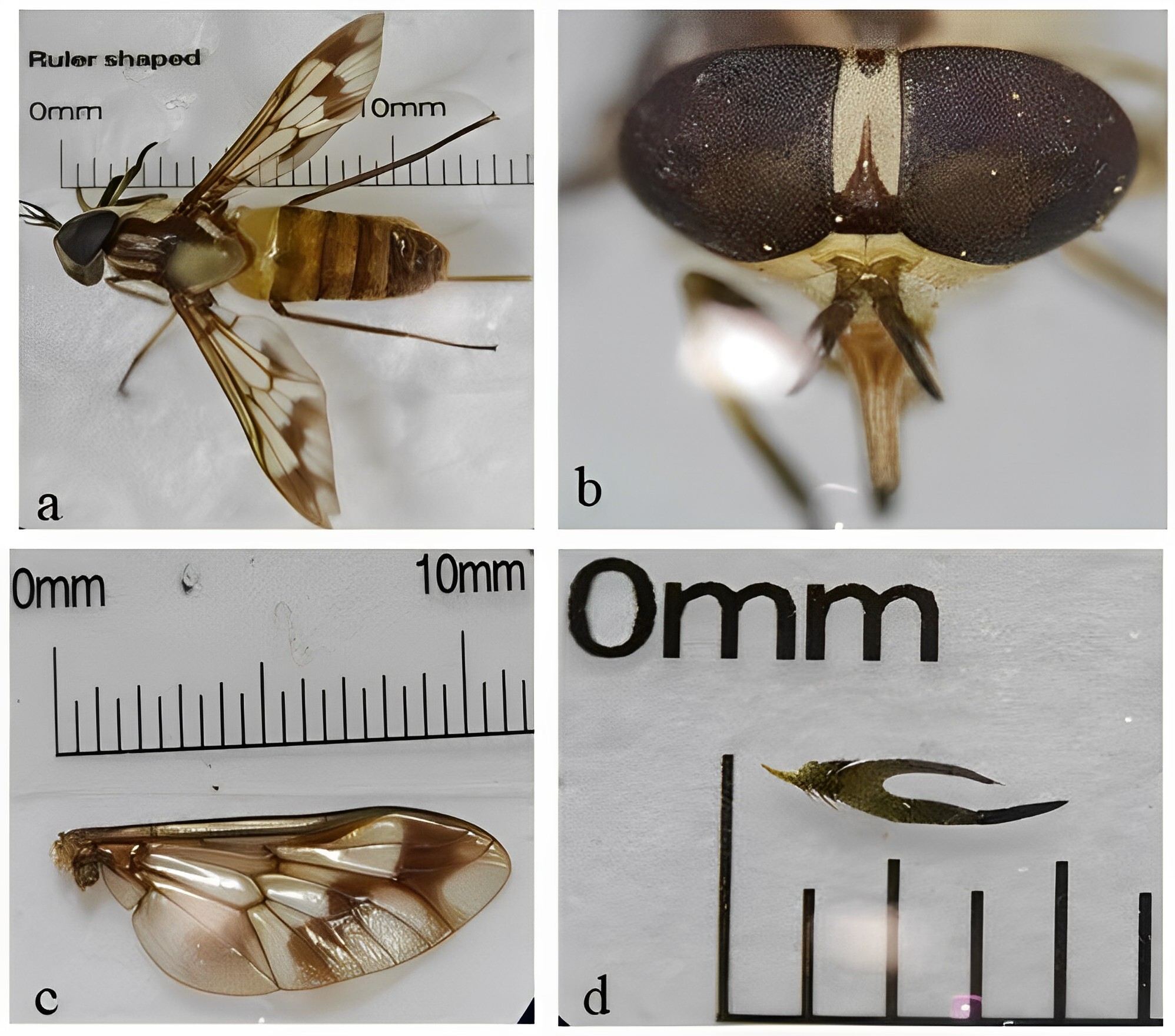

Supplement: Supplementary file 1 — Additional file 1: Figure S1. General morphology (a) and detail of head (b), wings (b), and antenna (d) of Dichelacera alcicornis. The scales are not the same in this composite figure. [file 13071_2022_5562_MOESM1_ESM.jpg]

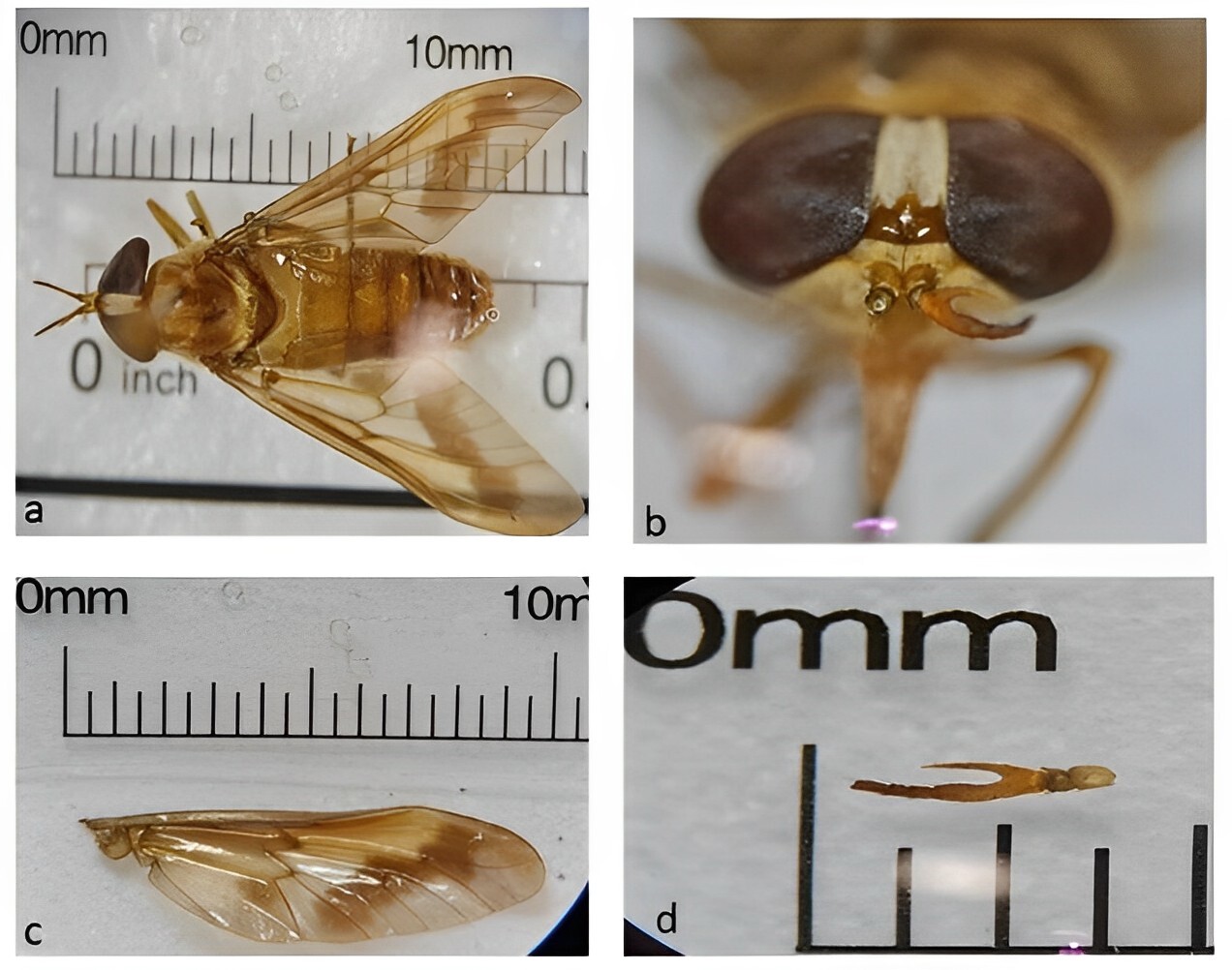

Supplement: Supplementary file 2 — Additional file 2: Figure S2. General morphology (a) and detail of head (b), wings (c), and antenna (d) of Dichelacera januarii. The scales are not the same in this composite figure. [file 13071_2022_5562_MOESM2_ESM.jpg]
